# Indigenous Border Migrants and (Im)Mobility Policies in Chile in Times of COVID-19

**DOI:** 10.3390/ijerph19159728

**Published:** 2022-08-07

**Authors:** Carlos Piñones-Rivera, Nanette Liberona, Rodrigo Arancibia, Verónica Jiménez

**Affiliations:** 1 Escuela de Psicología y Filosofía, Universidad de Tarapacá, Arica 1000000, Chile; 2 Departamento de Antropología, Universidad de Tarapacá, Arica 1000000, Chile; 3 Cooperativa Apacheta, Iquique 1100000, Chile; 4 Programa de Doctorado en Psicología, Escuela de Psicología y Filosofía, Universidad de Tarapacá, Arica 1000000, Chile

**Keywords:** healthcare mobility, borders, immigrant health, indigenous health, structural violence, structural vulnerability, social determinants of health, health inequality

## Abstract

The commodification of healthcare and the structural violence towards the migrant population in the Chilean system materialize in a series of structural barriers to accessing healthcare. In the face of this structural vulnerability, cross-border health mobility is one of the primary resources of indigenous border migrants living in the Tarapacá region (Chile). This involves crossing the border of both people (specialists/patients) and objects (such as ritual supplies or biomedicines), which play a crucial role as, in many cases, it is the only way to satisfy their healthcare needs. The security-orientated geopolitics of border closure (Plan Frontera Segura) has been reinforced by immobility policies linked to the COVID-19 pandemic. While doing so leaves people without the fundamental resource of healthcare mobility or obliges them to cross the border via unauthorized crossings, exposing them to criminalization and abuse by different agents of violence (the military, people smugglers, etc.). In this paper, we will offer a description of these processes of (im)mobility, analyzing their conformation both by the current policies of the Chilean State and by the notorious deficiency in indigenous and migrant rights, denouncing the material impact they have on the health/illness/care process of indigenous migrants.

## 1. Introduction

Neoliberal reforms to the healthcare system are linked to a worsening of collective health [1,2]. The commodification of all vital arrangements that is typical of neoliberalism in Chile has not only turned it into one of the countries with the highest out-of-pocket spending among the member nations of the Organization for Economic Co-operation and Development (OECD) [3,4] but also to have one of the highest costs for medications in Latin America [5]. The latter is due to the structure of a strongly monopolized pharmaceutical market focused on sectorial decisions rather than on people’s rights and needs [6]. Additionally, waiting lists for hospital treatment present serious problems: just between January and June 2018, there were 9724 deaths of people on such waiting lists [7]. In general, the system systematically pushes people into debt for basic subsistence, shaping a subjectivity that, in addition to suffering structural inequity, is held responsible for such suffering [8].

One phenomenon studied on the northern border of Chile is related to the journeys people make to neighboring countries to seek healthcare attention [9]. The literature indicates that this form of mobility is not carried out due to nomadic customs, nor for the pleasure of traveling. Instead, it is often motivated by the system’s inequity, based on a neoliberal model that strengthens the healthcare market and leads to a population segmentation according to income and risks [10], thus hindering most people’s access to quality care [1,2,3,4,5]. This problem becomes more acute when it affects migrant populations, who also suffer the compounded effect of phenomena such as cyclic irregularity [11], racism in healthcare [12], as well as other aspects included in the category of “Social Determinants” [13,14,15,16,17].

Although the studies of healthcare mobility have contributed new perspectives in understanding health/disease/care processes, we can observe two main limitations. The first has consisted of focusing restrictively on biomedical care while ignoring the contribution of other forms of knowledge that contribute to the medical pluralism of the current regions of Arica and Parinacota, and Tarapacá in northern Chile. Such is the case of Andean traditional medical knowledge, which plays a vital role for the Chilean and migrant population. The second is that although these works consider the relationship between mobility and territory along the lines proposed by Tarrius [18], they have not analyzed the role of medical knowledge in specifying how it actually circulates. Thus, circular knowledge appears as an abstract and unspecific term in these studies.

Our research questions are: what are the processes of (im)mobility that affected the health mobility in the Tarapacá region (Chile) in COVID-19 times? How does this (im)mobility process impact the health/illness/care process of indigenous migrants? How do the current policies of the Chilean State and its deficiency in indigenous and migrant rights give form to that (im)mobility process?

**Figure 1:**
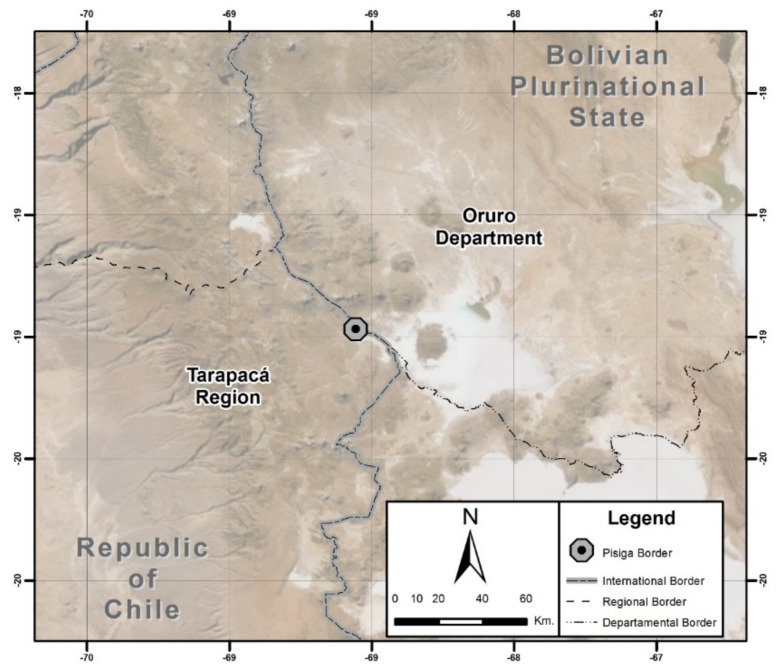
**The** Chile−Bolivia cross border region.

Our main argument is that the security-focused geopolitics of the border closure (Secure Border Plan) [19] has been reinforced by COVID-19 immobility policies. Those policies leave people without the fundamental resource of healthcare mobility or oblige them to use unauthorized border crossings, thus exposing themselves to criminalization and abuse from different agents of violence (such as people smugglers or the military) [20]. For this purpose, we will focus on what happens on the Chile-Bolivia border (Colchane) (see Figure 1). We wish to offer a description of these processes of (im)mobility, analyzing their conformation both for the current policies of the Chilean State and for the notorious absences in the area of indigenous and migrant law, revealing the material impact they have on the health/disease/care process of indigenous migrants.

### 1.1. (Im)Mobility Policies in Border Studies

#### 1.1.1. Thoughts on (Im)Mobility

Border studies are increasingly interested in the interaction and links that occur through them and that people deploy in their constant crossings [21,22], which is how mobility takes a prominent place in the analysis. These studies question the generalized use of the notion of migration because it prevented the capture of some population movements. Heyman [23] posits that mobility makes “fewer judgments about the nature of the movement (duration, purpose, social position)” (p. 427). Tarrius, for his part, developed a paradigm of mobility based on the space-time-identity triad within the framework of the anthropology of movement, which allows “understanding how crossings of space are always also crossings of social hierarchies” [18] (p. 45). He thus posed that any “social, cultural and economic mobility leaves a mark in space and time” [18] (p. 45). Based on an analysis of migratory circulations (a notion derived from French social geography), as evidenced in his studies on the trade between Marseille, Algeria, and Morocco [24], this author identified the convenience of the notion of circulatory territory, which “confirms the socialization of spaces according to logics of mobility” [18] (p. 55). From these perspectives, border regions are understood as the right place to capture the variety of movements and to observe them from a territorial perspective [25].

Due to the discussion regarding spatial mobility and territory, an interest arose concerning its counterpart, as immobility allows mobility to be visualized. Following this argument, we refer through processes of (im)mobility to the mobility/immobility continuum as a dialectical relationship in the production of space, as well as a relational experience, differentiated and located in geographical and historical terms, which is a consequence of the unequal exercise of power [26]. The authors point out that mobility cannot be understood without immobility because they are dialectically related and determine each other. We adopt this concept from critical human geography, as it allows us to realize that the movement of people is an embodied experience that produces and reproduces space.

Nevertheless, contemporary border studies consider both the mobility of people and that of goods, capital, and information [27], while the border, for its part, functions as a filter or system of differentiated flows [28]. In this sense, Heyman’s theoretical contribution is relevant, given that by stating that there is unequal mobility [27], he introduces the analysis of border control policies as mobility control policies. Heyman explains that unequal mobility is understood from two fundamental points: firstly, it occurs in contemporary capitalism (with a connection between unequal mobility, borders, labor, and capital); and, secondly, it is the result of a complex relationship between policies and the functional needs of capitalism.

Thus, the cross-border space that we observe in this work results from the (im)mobility produced in this case by people subject to forced displacement, border migration, and objects that circulate in relation to Andean medical knowledge. However, the (im)mobility processes are determined by border and migratory control policies that we will present below.

#### 1.1.2. Securitization and (Im)Mobility Policies

As we have mentioned, the processes of (im)mobility are the focus of policies that seek to control the borders and the various movements across them. These include migration, interpreted as a security problem for states. The international literature has identified securitization as one of the main trends pertinent to these policies. As such, it is associated with the political establishment’s fear of losing symbolic control over their territorial limits and has been structured by the habitus of security professionals and by the globalization of surveillance technologies. Ultimately, however, securitization is based on the discomfort of citizens who feel they cannot deal with the uncertainties of everyday life in a neoliberal society [29]. Other theoretical approaches define securitization as a response to migration perceived as a political-identitarian threat [30,31] or a socioeconomic thread [32,33].

Such security-focused policies view migrants as dangerous subjects, and one of their manifestations is the criminalization of such people [34]. The Chilean approach to migration adopted the figure of the migrant as the enemy within the State during Pinochet’s dictatorship [35]. However, securitization is far from just a Chilean phenomenon and responds to a global trend [36,37]. Eduardo Domenech [38] proposed that South American immigration policies have turned towards a “punitive approach” in recent years, intensifying state violence towards migrants in a context of essential changes in migratory patterns. Examples of these changes are the militarization of the Peru-Ecuador and Chile-Bolivia borders in 2020 to control and limit mobility. Likewise, the imposition of visas to Venezuela from 2019 by Ecuador, Argentina, and Chile. These trends are adapted to the creation of a regime of global migration control, characterized by “the emergence of new ways of thinking and acting on migrations such as migration management” [38] (p. 22), the purpose of which is the regulation of international migrations in a neoliberal global framework. These policies focus on preventing, eliminating, and regulating “migratory pressures”. One of the culminating instances of this global regime is the Global Compact for Migration (2018), which assumes migration as “safe, orderly and regular”. After analyzing the narratives of international agencies, Pécoud affirmed that the use of the term migration management is reassuring; “a managed phenomenon is a phenomenon under control, which will probably not escape the regulatory capacity of States”. [39] (p. 22). In this context, migrations are conceived as a positive phenomenon that can benefit multiple stakeholders if adequately administered. This positive image conceals the structural vulnerability that directly affects people in movement.

## 2. Materials and Methods

The data analyzed for this research has resulted from collaborative ethnographic work, focused on socio-legal and health support for migrants who reside in or transit through the Tarapacá region (Chile) and who have encountered the leading organization dedicated to the defense of the human rights of migrants in the said region: the Open Assembly of Migrants and Pro-Migrants (Spanish acronym, AMPRO). Such support in their migratory procedures and access to social rights represent the main instances in which observation of participants is carried out and field notes collated. Collaborative work was fundamental for establishing a connection with the people and for the team’s conformation, including a migrant facilitator and leader.

In our current research project, the main stakeholders are people requesting refugee status, who are residents or are in transit through the cities of Arica, Iquique, and Alto Hospicio, and who entered Chile via border crossings in the regions of Arica and Parinacota, and Tarapacá (Table 1).

To compare the information gathered from participants with that of local and national institutional actors, we conducted semi-structured interviews with key informants, such as local authorities and technical personnel, to understand how migration and border policies are interpreted and applied. Other key informants were members of NGOs (such as the Red Cross, Jesuit Migrant Service, and UNHCR), along with social organizations that provide legal and social assistance to this population segment (Table 2). We also made observations at the frontier crossings in the regions of Arica and Parinacota, and Tarapacá.

In addition, we conducted a documentary analysis of various related national and international regulatory bodies and reviewed requests for official documents through the Chilean government’s Transparency website, which provides access to public information. We also monitored the local, national, and international media regarding the border closure due to public health concerns.

The study was conducted in accordance with the Declaration of Helsinki and approved by the Institutional Review Board (or Ethics Committee) of Universidad de Tarapacá for studies involving humans. Informed consent was obtained from all subjects involved in the study.

## 3. Results and Discussion

### 3.1. Cross-Border (Im)Mobility Processes in Times of COVID-19

The COVID-19 pandemic led to a state of exception in Chile, which included border closures (Supreme Decree 104 of 18 March 2020). However, it is possible to describe a continuity between said closures and other border policies related to migratory control and the control of illegal activities. Just as a political and media link was developed internationally between migration, organized crime, and terrorism [40,41], in Chile, migration was consistently associated with drug trafficking and smuggling [42,43,44].

The link-up between these three aspects clearly manifests the securitizing tendency that has permeated Chile’s immigration and border policies. This focus on security has impacted the process of (im)mobility, strengthening the restriction of people’s movements. A clear example of this is the package of measures announced by former President Sebastián Piñera on 23 April 2018, which included a migration bill, an anomalous migration regularization process, increased entry restrictions for Haitians through the imposition of a consular tourist visa, the creation of a Democratic Responsibility visa, exclusively for Venezuelans, the elimination of the temporary work visa, the strengthening of mechanisms to carry out expulsions and the implementation of the Secure Border Plan in the five regions of northern Chile. It is essential to highlight that the focus on certain national groups (especially Haitians and Venezuelans) shows that these are racist-influenced policies, as we have analyzed in other works [45].

A speech introducing this law, made by former President Piñera, clearly shows its criminalizing characteristics:

The main objective of this new law is to Put our House in Order through an orderly, safe, and regular migration policy, which allows legal migration and combats illegal migration […] those who enter or try to enter our country clandestinely by unauthorized means, not only commit a crime, they also risk being subject to expulsion.

[46]

As Dufraix et al. [19] have shown, the result of these policies, adhering to the concept of “ordering the house”, have not led to orderly, safe, and regular migration; instead, they have increased migratory irregularity. For example, the Democratic Responsibility Visa (DRV) presented by the government of Sebastián Piñera in Cúcuta only granted 27% of all applications up to January 2021. Moreover, the Consular Tourism Visa (CTV), which came into force in 2019, has only been issued for 21% of all applications up to January 2021 [47].

The same is also true of the asylum law. Celebrated as a law drafted to the level of international standards, it has been neutralized by the political decision to make it inapplicable [48]. Proof of this is that in 2019, having registered 16,748 requests for asylum before the Chilean border authority by Venezuelan nationals, the Chilean State recognized the refugee condition of only five such citizens. In 2020, only seven Venezuelans were recognized as refugees after 1629 applications were officially processed [47]. In 2021, the processing of asylum applications decreased considerably due to a policy of disincentives and access barriers, as revealed by the Comptroller’s Report on the closure of this process by the country’s border authorities for the year 2021. Through Decree 7.196 of 2021, the government instructs to suspend, in certain provincial governorates, the reception of applications for recognition of refugee status under Law 20.430. Likewise, “State agents have hindered the possibilities of persons seeking international protection to claim the right to request asylum” [48] (p. 408).

Lastly, and shortly before the Piñera government left office, the Ministry of Interior and Public Security ordered a modification to the Regulation of the Law of Asylum (Decree 125), imposing a pre-admissibility measure, which was previously used arbitrarily, to limit the processing of asylum requests, thus institutionalizing this practice.

One interesting aspect is that these securitizing measures have adopted the language of migrant protection, concerning the dangers inherent to people trafficking networks and “the risks derived from such people’s irregular situation in the country” (paragraph 3 of decree 776 of the Ministry of Interior and Public Security). However, as Huysmans and Squire [49] have analyzed, such a humanitarian discourse is part of the securitizing strategy, hence its dual nature.

In the case of the border with Bolivia, which we analyze in this article, the principal measures established have been restrictions on mobility. However, as we will analyze further, such measures then acquired a new characteristic and legitimacy concerning the processes of (im)mobility propitiated by the COVID-19 pandemic.

#### 3.1.1. (Im)Mobility Policies Linked to the Pandemic

Immobility acquires its meaning in a broader political context when promulgating a state of exception. It confers special powers to the State, placing limits on the exercise of rights, for example, those of mobility. However, the fact that rights are limited is not a problematic issue in itself. The basis of rights’ limitation is the need to reconcile the exercise of each right with the protection of certain valuable assets for the greater good or with the exercise of the rights of third parties. What is problematic is the identification of these limits and the scope of the restrictions allowed by the legal system. In the case of pandemics such as COVID-19, states can limit the exercise of rights to protect public health. Moreover, unlike what happens with other protected interests by which rights are limited, such as national security, public order, or public morals, in the case of public health, criteria and technical reasons customize more defined and objective profiles applied in the context of migration control [50]. Thus, the closure of borders generates an unquestioned consensus in the context of a pandemic, overshadowing political processes such as the securitization or criminalization of migration, actions that then experience a previously controversial legitimacy.

On 17 March 2020, through Decree 102, the Chilean government ordered the temporary closure of borders due to the presence of cases of COVID-19. The argument was the “health alert for a period of one year […] due to a public health emergency of international importance, caused by the outbreak of the new coronavirus”. According to article 57 of the health code, “adequate measures must be established to prevent international transmission”. All of the above occurred because “the State must protect the population, as it is responsible for coordinating and controlling actions related to the population’s health”.

However, borders are never an absolute barrier but rather an exercise that transforms the mobility of people into politics to decide how and who can effectively cross them [51]. The regulations segmented entry into the country based on specific criteria: the entrance route (land or air); nationality (Chilean/foreign); immigration status (foreigner with permanent residence/undocumented foreigner/foreigner with exceptional credentials, such as diplomatic visas); family links (with children or parents resident in Chile/or without them). Thus, the border policy excluded people who entered by land routes, did not have special documents, and had no resident parent or children in Chile. This control was strictly applied in the terrestrial border crossings and much more flexible in the aerial entry points, which indicates a class bias in its implementation.

In this scenario, it is essential to clarify that the conditions for traveling to the country with a resolved migration situation were restricted. Thus, the publicized Democratic Responsibility Visas (in force since 2018) were suspended in November 2020. According to the information given to applicants, this was attributed to the sanitary closure of Chilean borders, “due to which the completion period of the administrative process has been surpassed”, referring to the Democratic Responsibility Visa [52]. This was how the massive rejection of such visa applications was justified. The possibilities of formalizing their migratory status once inside the country were also tricky. Indeed, eliminating the work visa meant that many people who had entered as tourists could no longer formalize their situation.

**Figure 2:**
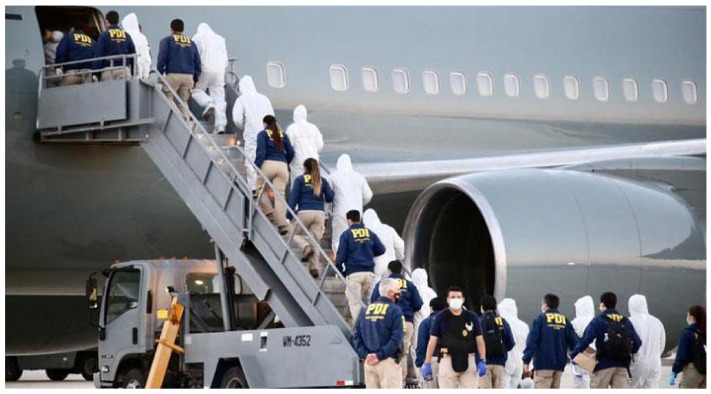
Expulsion of 138 foreign nationals under the Colchane Plan.

However, the most shocking occurrence in terms of media exposure was the illegal group expulsions that heralded the so-called “Colchane Plan”. Almost a year after the border closure, inter-institutional coordination was established to implement the said plan, the purpose of which was to counter the increase in the number of illegal crossings through the Colchane Pass on the Chile-Bolivia border. Within this context, and between 3 and 8 of February 2021, four expulsion orders were issued that led to the deportation of 138 foreign nationals (Figure 2). The expulsions consisted of authentic cases of media staging that, through criminalizing migration, increased the hostility of the Chilean population towards migrants while justifying the illegality of the actions carried out by the Chilean State, given that the expulsions were massive and did not adhere to legal standards of due process.

According to the analysis carried out by Jiménez [53], in none of these resolutions was there an individual, objective and rational evaluation of the circumstances of each person (criminal records, their previous background in their countries of origin, or any analysis related to the family or working roots of the people, or any deliberation of people’s welfare related to the above). This violates the principle of non-refoulment, which corresponded in most cases to Venezuelans expelled from Chile, the vast majority of whom represented cases of forced migration, thus meeting the conditions to receive refuge according to the Convention relating to the Status of Refugees of 1951. Furthermore, there was no guarantee of access to an appeal against the expulsion order, as the period to lodge such a plea was 24 h, which made it completely unviable, given that the court specified for the procedure was the Supreme Court of Chile, located in the country’s capital almost 2000 km away.

Conceptually, all these measures were intended to prevent the international transmission of the coronavirus. However, the securitization criterion absorbed the health criterion, as we can conclude from some specific situations related to health. One of the most significant in this regard was the requirement of voluntarily reporting to the Investigations Police (PDI) to access preventive quarantine for all people who entered the country using an unauthorized route. Consequently, a registry of those who entered by irregular means was established, while it was unclear what the purpose of this procedure was.

While some people were told that this would serve them in any subsequent regularization process, the authorities also announced expulsing those who had entered by such irregular routes. Consequently, many people avoided going to the healthcare-quarantine residences for fear of being exposed to expulsion. Moreover, this was carried out according to a rationale of minimally sufficient healthcare provision. That is to say, once people received minimal care, the next step was their expulsion.

Another example in which the security objective became paramount to the health objective can be observed in the initial approach regarding the vaccination of undocumented foreigners. Indeed, the first instruction was that migrants with tourist visas and those that were undocumented would not be vaccinated. This instruction provoked strong criticism from migrant and pro-migrant organizations, which finally reversed said directive.

Finally, the policy to close the border led directly to an increase in irregular crossings, thus encouraging the criminal activity of the so-called “coyotes” or people smugglers, who profit by aiding people to cross the border. This policy leaves migrants in a highly vulnerable situation, and many have suffered abuse from smugglers or even lost their lives [20].

As can be seen in these examples, although the State of exception was based on sanitary reasons, the political decisions often subordinated health reasons to securitization.

#### 3.1.2. Militarization for Public Health

Another aspect worthy of analysis of the Colchane Plan is related to the process of militarization that accompanied it. On 4 February 2021, the Chilean government, through a modification of decree 265, authorized the Armed Forces to assist the police in matters of migration to protect the borders through technology and patrols.

In addition to the media staging of the expulsions analyzed above, the militarization of the borderlands generated impacts on local indigenous communities. They, in turn, denounced the discriminatory treatment they were receiving, as stated in the application for protection corresponding to Court Inscription No. 21-2021, presented by Adimelia Moscoso, Elías Mamani, and Bárbara Montecino:

[The army] frequently confuses Chilean Aymaras with Bolivian Aymaras, treating them with discrimination related to identity control and the native language of the Aymara people. In this sense, there is no equality of rights regarding movements between Chilean and Bolivian Aymaras, concerning the hundreds of migrants who cross the border, in which it can be observed that they are not subject to any control.

[54] (s/p)

Furthermore, it also hampered cross-border mobility between Chile and Bolivia, typically undertaken by members of indigenous communities:

Aymara communities, in this sense, have been prevented from accessing a series of products they exchange or get a reasonable price for in surrounding communities. This has increased the cost of living of Aymara families and led to a shortage of specific products such as gas, non-perishable food, and coca leaves, among others, which are vital for our people. In addition, they have prevented members of the community from going to the aid of family members, including the elderly, who in the communities have a fundamental role as they are custodians of the knowledge and wisdom of ancestral uses and customs. The high level of administrative surveillance of ‘official border crossings’ has caused a greater flow through ‘unauthorized’ crossings. Our communities have seen an increase in the frequency and quantity of vehicles (including caravans of up to 40 vehicles), sometimes related to drug trafficking, smuggling, and other crimes”.

[54] (s/p)

This was reflected in comments made by the Mayor of Colchane, who stated as follows:

The militarization and placement of two armored vehicles on the border, along with military personnel, has only controlled our people, not the immigrants, as the army provides humanitarian assistance to migrants […]. I believe that what is needed here is some self-criticism and to sit down and get to work, as it is not just this issue of the humanitarian point of view of the rights that migrants have to migrate, but also the rights that we have, for example, the indigenous peoples, as we deal with this migratory phenomenon, which is not our responsibility, and that is clearly detrimental to our cultural identity.

One clear example is the issue of coca leaves. These leaves play an integral role in the life of the Aymara as food as well as medicine (in its ritual aspects and chemical properties); this is a sacred plant used in the offering rituals made to the Pachamama and protective beings. However, it is not grown in Chile, so it enters the country mainly from Bolivia and Peru. Although it crosses into Chile via unauthorized routes, thus avoiding border controls, officials of the Agricultural and Livestock Service (Servicio Agrícola y Ganadero-SAG) and customs officials, who understand its importance, allow the entry of small amounts of leaves for personal consumption.

In the context of the closure and militarization of Chile’s borders, this criterion has been suspended, as those bringing in coca leaves are obliged to use unauthorized routes, thus exposing people to having to explain themselves to the military, who do not possess such an understanding of the customs specific to the territory. Moreover, neither have soldiers been trained to use flexible criteria when dealing with a heavily criminalized plant. Such criminalization has a long history, led by the United States, which, based on the Geneva Convention (1936), obliged other states to pursue drug trafficking and any actions involving “harmful drugs”. Furthermore, an essential step in the criminalization of coca leaves was their inclusion in the Single Convention on Narcotic Drugs of 1961 of the United Nations (1961 Convention), modified by the Geneva Protocol in 1972, signed by 74 countries [55].

Thus, the movement of vital elements for the region’s indigenous communities, such as the entry of coca leaves, was affected not only by the closure of borders but also by their consequent militarization, generating an inadvertent impact on health. To understand this impact, we must consider the existing medical pluralism, specifically the role of so-called “traditional” knowledge. Of course, the value of such knowledge is not restricted to the native people’s or neighboring countries’ populations. As we have shown [56], it also constitutes one of the primary resources for the undocumented migrant population.

Paradoxically, this impact on health was implemented based on legal arguments regarding health. What happened is that, in the context of COVID-19, the health domain was reduced to the coronavirus domain. Thus, the monocultural, biomedical approach of the State once again shows its limitations. However, to get a broader idea of the impact on health caused by such immobility policies, it is necessary to leave aside the monocultural rationale and observe the impact they had from the point of view of Andean medical knowledge.

### 3.2. Impact on the Health/Disease/Care Process

I traveled first… the first year I was making the journey every three months, because as I did not have a visa, so I had to leave no matter what, because they told me: you have to have your papers in order to be able to get the visa, so you cannot have anything out of order, things like that. Then, I went every three months, and I saw them and came back again. I entered […] I was almost a month, and then I returned. […] The last time I left was just before the pandemic… and then a month went by, and just when the pandemic was beginning, I returned. That month that we came, the pandemic had started, we had already entered, and I could not leave… (N., Bolivian woman, interviewed in May 2021, Iquique.)

The mobility of people was affected, including healthcare mobility. As we have shown in previous works [56,57], cross-border healthcare mobility is a frequent and essential socio-spatial practice in Tarapacá. This mobility does not consist only of searching for patented drugs or biomedical attention. It also includes the search for supplies such as medicinal plants, naturist preparations, and products made by the respective domestic producers based on local herbal remedies. Furthermore, an essential part of health mobility is to provide elements related to the healing practices of Andean medical knowledge [57].

Since time immemorial, Andean medical knowledge has drawn on the different ecological foundations. For this reason, it has been and is transnational and cross-border in nature. The origin of its inputs shows a complex interrelation of legal spaces and regimes. Thus, the elements sold for ritual ‘mesas’ or sacred offerings in the territory of Tarapacá come from Oruro, while many of the naturist products are sourced from the national industries of Peru and Bolivia. Medicinal plants come from the different ecological floors present in these three countries.

Moreover, the ways that they enter the country also vary. According to a Chilean customs officer, most likely, the bulk of these products enter the country thanks to small-scale smuggling. They use the so-called “traveler franchise” that allows a person to enter the country with everything in their luggage. Thus, when someone wants to introduce a more considerable volume of products, they are distributed among people paid to carry them as part of their luggage [58,59]. Another modality is the payment of bribes to officials to let contraband trucks pass through the border checkpoints. Some Bolivian informants said they pay about US$5000 for this purpose, making a collection among all those interested in the truck coming across the border.

These strategies of transporting therapeutic goods pass through various regulatory spaces [60], of which the dominant one is that of the State. Within the framework we are analyzing, this means that the assets of Andean medical knowledge are, in general, criminalized objects. And this occurs either in their origin (when their production is prohibited), in one of the intermediate links by which they circulate (when the prohibition falls on the transfer from one country to another), or in their sale. The criminalization, illegalization, and State delegitimation of Andean medical knowledge is also applied to health and knowledge agents [61,62]. However, this does not prevent such agents from enjoying an important level of appreciation in the popular sectors, occupying a place characterized by the coexistence of legal rejection with the appraisal offered by social actors [60].

And while these networks are part of globalized processes, they take place within a context of strong historical roots in the Andean economic space [63], where indigenous people produce most of the goods sold. In addition, their flow through the commodity chain until they reach the consumers passes through various regulatory spaces where ethnic and kinship networks are indispensable for evading state control [64,65,66].

Thus, a significant impact is related to the difficulty of legally crossing the border to acquire supplies for treating health problems in adherence to an Andean rationale.

Z.A.: So, whenever I travel to Bolivia, people in Chile ask me to bring back medicines, all from there. […] I brought ovules for body pain, oil, and natural medicine. What they have confiscated are the maticos, the dry leaves. I’ve brought a lot of that, and they have confiscated it […] I had about seven herbs, including chamomile, fresh chamomile, and here there is not any… the same with muña, and cola de caballo. There are two types of cola de caballo, one that’s yellow and the other green. I brought the yellow one. I also brought boldo.

C.P.: Ah, and you brought them from Cochabamba?

Z.A.: From Cochabamba, without the stems. Just like they sell parsley, all tied together, just like that. (Z.A., Bolivian woman, interviewed in September 2020, Iquique.)

However, just as these anecdotes show the direct impact of the processes of (im)mobility in the practice of self-attention, a more significant impact can be observed in the obstacles imposed on the movements of traditional healthcare practitioners such as Yatiris (spiritual healers), Qulliris (bone setter and herbalists), and Usuyiris (traditional midwives).

These processes of outlawing such movements have been exacerbated in the context of (im)mobility caused by COVID-19. To illustrate this point, we find it appropriate to present the story of E.C., a Bolivian healer from the outskirts of La Paz (Bolivia) who now lives and works in Alto Hospicio (Chile). He reminds us that the specific nature of Andean medical knowledge requires that certain ingredients must be brought from beyond the borders: “Misa de rayo con tata Santiago [specific type of ‘mesas’ ritual offering] has to be carried out… and it does not exist here. Well, it exists, it is not that it is not here, but it is not my way of doing it, another way”, as he well exemplified for us. Such practices are not taken into account by border control agents who, by preventing the open transit of such medicinal elements, force them to be transferred by illegal means. This situation has worsened since the implementation of the Secure Border Plan.

E.C.: They wanted to confiscate that from me… in an apple box, you can fit 25 units of large ‘mesas’ offerings and about 40 little ones. When I was inspected by the SAG official [he said]: ‘I am letting you go now, but sign this other form, do it for me. And if you carry this stuff again, I’m going to arrest you’ […] Last year it was not so bad… then I would bring about 2 or 3 crates of apple boxes, […] brought them on the bus without any hassle. But now [since the border closure], it is just not on. You have to break the law and bring it all in illegally. (E.C., Bolivian yatiri healer, interviewed in July 2020, Alto Hospicio).

Despite what the case of E.C. shows, such flows continue from Chile to Bolivia. In general, control surveillance has focused on the dynamics of entry into Chile and mainly on the Venezuelan population due to their country’s humanitarian “crisis”. However, other permanent flows of people are associated with seeking attention for health problems and seeking options other than biomedical treatments, which have been systematically neglected.

**Figure 3:**
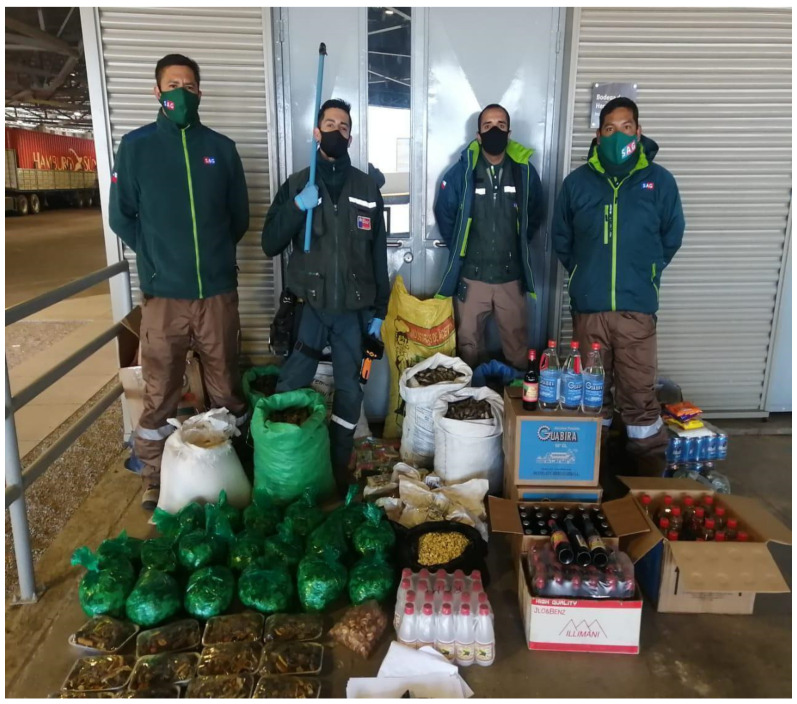
Confiscation of Bolivian truck cargo, including coca leaves, ‘*mesas*’ offerings, and ‘*purito*’ (90% proof alcohol), among other goods. Source: Agricultural and Livestock Service-SAG, September 2020.

One example of these flows and the validity of Andean medical knowledge was the seizure made by the Agricultural and Livestock Service (Servicio Agrícola y Ganadero-SAG) on 2 September 2020, of a truck from Bolivia passing through the Chungará border control post: the truck’s load consisted of 200 kg of “forestry and agricultural products” that included “sacks of coca leaves […], 74 L of alcohol for consumption purposes, and ten alpaca fetuses, the latter being quite unprecedented in the region and representing a high risk to public health” [67]. The confiscation also included black ‘mesas’ offerings (Figure 3), omitted in the official statement and newspaper reports. The director of SAG, Jorge Hernández, made the following remark:

… This type of cargo, specifically the alpaca fetuses, which are domestic South American camelids, represent an extreme risk to domestic (animal) health, as they can carry exotic diseases not present in the country, which could have a considerable impact on domestic production and affect the export of Chilean products to critical foreign markets.

Curiously, the presence of alpaca fetuses in a region where these animals are widespread is described as “unprecedented”. Also, curious seems to claim a high risk to animal health in a border zone where livestock transport from one side of the border occurs regularly. Moreover, when the cross-border networks of the popular economy make these elements available daily for the ritual and health needs of the population. Nevertheless, this is how the border control practices of the Chilean State have fostered the “criminal life” [60] of essential objects of Andean medical knowledge.

### 3.3. Deficits in Indigenous and Migrant Law

Chile has signed and ratified numerous human rights instruments. The so-called constitutionalization of the different branches of law enables the direct application of such treaties under the provisions of the second paragraph of article 5 of the Political Constitution of the Republic, either as self-executing norms or as norms that serve for the systematic or organic interpretation of Chilean laws. However, in many cases, the implementation and exercise of these rights are subject to the adaptation of internal regulations, which in monocultural structural contexts is challenging to implement. Thus, the immobility processes reported in this article are supported by significant deficiencies in the domestic law for migrant and indigenous populations.

Human rights represent the backbone of international law; thus, the need to prevent discrimination and abuses suffered by migrants is a long-standing concern that has been led by international bodies, with the right to freedom of movement and to choose a place of residence expressly recognized in the Universal Declaration of Human Rights (1948) and the International Covenant on Civil and Political Rights (1966).

At the regional level, regional integration initiatives, such as the Southern Common Market (MERCOSUR), have established free movement agreements, particularly regarding border areas. Bolivia and Chile are associate members of MERCOSUR, through their respective Economic Complementation Agreements, and must therefore respect the Agreement on Residence for Nationals of the MERCOSUR States Parties, Bolivia, and Chile. The latter involves facilities for the movement of workers in border areas. In the field of health and border, an important step was taken with the signing of the 2019 Agreement on Linked Border Localities [68]. However, the assessment of access to biomedical health in MERCOSUR border areas in pandemic contexts is rather negative. Considering all States opted to prevent the spread of the virus contagion by closing borders, a rebordering effect was produced [69], limiting interaction at the borders, mainly affecting local communities. The PAHO has expressed concern because these areas are home to vulnerable populations, including indigenous people, communities living in remote areas, and immigrants [70]. Regarding indigenous rights, given the institutional structure of MERCOSUR, the contribution of this instrument depends on the recognition that each State party or associated State gives to its indigenous peoples; and Chile still has not recognized indigenous peoples’ existence in its constitution [71].

Considering all the above, the main effects on migrants relate to the right to admission to Chile, the right to legalize their residency status, the right to receive refuge, the right to non-return to their country of origin, the right to due process concerning their humanitarian situation and the right to receive healthcare without discrimination. In this case, the adverse effects and the dramatic impacts on health arise from the primacy of a security-orientated form of being treated, particularly regarding the medical treatment associated with COVID-19. Thus, appropriate measures have not been witnessed that would allow migrants to avoid the risk associated with their vulnerability, especially in the case of children, or to avoid being exposed to acts of xenophobia, discrimination, and stigmatization.

In the case of native peoples, it must be taken into account that specific rights enshrined in international law refer to cross-border mobility. Ultimately, these rights are based on the right to cultural integrity, the right to culture, and cultural heritage. The first thing to point out is that Supreme Decree 104, which declares a state of constitutional exception of catastrophe and allows the restriction of certain rights, has ignored the right to participate in measures that directly affect peoples and communities. Consequently, the measures implemented to protect the security and life of citizens lack any sense of interculturality, given that regulations emanating from the central level are established, with total ignorance of the regional territorial dynamics of native peoples and cross-border existence.

Regarding healthcare, this has led to a setback in the dialogue held with the State. Just to give an example, during the process of participation and subsequent indigenous consultation of article 7 of Law 20,584 (a process that lasted for more than five years), the representatives of native peoples of the whole country informed the Chilean State of the necessary conditions to guarantee the right to healthcare with cultural relevance, pointing to problems such as being obliged to use practices of the biomedical model, among many others. For each problem identified, proposals were drawn up by consensus between the State and the different native peoples in a detailed document submitted to the Office of the Comptroller General of the Republic. Today, this entire process of dialogue has not only been ignored but has been made entirely imperceptible, as if the government had to provide solutions to the pandemic without any previous guidance in this regard.

In terms more directly related to cross-border mobility, international regulations have made progress in establishing, as stated in the American Declaration on the Rights of Indigenous Peoples, the right to association, assembly, organization, and expression and to exercise them without interference. Furthermore, it explicitly guarantees the right to “travel and to maintain and develop contacts, relations, and direct co-operation, including activities for spiritual, cultural, political, economic and social purposes, with their members and other peoples” (Article XX). Although Chilean legislation has not been aligned with the American Declaration (ADRIP, 2016), the ILO Convention 169 of 1989 has been ratified, which, being a more limited instrument in terms of standards, establishes that governments must “take appropriate measures, including by means of international agreements, to facilitate contacts and co-operation between indigenous and tribal peoples across borders, including activities in the economic, social, cultural and environmental fields”.

Mobility for healthcare purposes represents a process directly associated with what is established by law, as it consists of economic activities (exchange, sale, consumption), as well as those of a social and cultural nature, due to the symbolic and ritual activity that is inherent to them. Likewise, it comprehends environmental activities, as reciprocal relationships with the territory are cultivated, taken care of, and reproduced in the environment and communities.

Furthermore, Andean medical knowledge constitutes an essential part of the cultural integrity of the indigenous peoples of this territory. In this regard, Convention 169 established that “Governments shall have the responsibility of developing, with the participation of the peoples concerned, coordinated and systematic action to protect the rights of these peoples and to guarantee respect for their integrity”. The aforementioned right is specified and expanded in more advanced formulations such as the United Nations Declaration on the Rights of Indigenous Peoples or the American Declaration, which establishes the right to “their own cultural identity and integrity and to their cultural heritage”, whether this is tangible or intangible, and not limited to the present, as it includes historical and ancestral heritage (Article XIII).

In the case of Andean medical knowledge, we are in the presence of an institution that is key to maintaining cultural integrity [72], as it maintains an essential part of the cosmovision, spirituality, and the relationship with the territory. This knowledge is embodied in institutions, practices, beliefs, and values. Likewise, agents with this knowledge represent carriers of a tangible and intangible cultural heritage. Thus, neither they nor their activities should be persecuted or criminalized but protected.

In this sense, the American Declaration states: “Indigenous peoples have the right to recognition and respect for all their ways of life, cosmovisions, spirituality, uses, customs, norms, traditions, forms of social, economic and political organization; forms of transmission of knowledge, institutions, practices, beliefs, values, dress, and languages, recognizing their interrelationship as established in this Declaration” (Article XIII). This right does not concern only to “protection, preservation, maintenance”, but rather the “development of that cultural heritage for their collective continuity and that of their members and so as to transmit that knowledge to future generations” (Article XIII). Therefore, protecting and developing indigenous culture is fundamental, as cultural continuity correlates with good mental health, lower suicide levels, and lower school dropouts in indigenous communities [73].

Although the limitation of rights is inherent to the legal system, the problem can be redirected to the fundamental aspects on which the rights are limited. Referring to the limitation of mobility in the COVID pandemic, one of the main problems is that the State operates according to a monocultural rationale. This rationale is rooted in the structure of the State and hindered the implementation and adaptation of internal regulatory frameworks to comply with international standards fully. Consequently, the rights of native peoples related to cultural integrity and cross-border mobility were not included in the design of the proposals implemented. As a result, a paradox was conformed in which measures taken in the name of health end up producing negative impacts on the population’s health.

## 4. Conclusions

Three questions guided our research: What processes of (im)mobility affected the health mobility in the Tarapacá region (Chile) in COVID-19 times? How did this (im)mobility process impact the health/illness/care process of indigenous migrants? How do the current policies of the Chilean and its deficiency in indigenous and migrant rights give form to that (im)mobility process? Regarding these questions, we want to highlight some ideas that have emerged from our work.

The immobility processes reviewed can be summarized in two groups, depending on whether they respond to processes determined by the immigrant condition or whether they operate independently of this condition. For the first set, we show how the (im)mobility processes were forged in continuity with racist immigration policies oriented towards security. In this framework, the apparent rationality of the health measures served as a legitimizing element of security policies, several of which were directly discriminatory and contrary to law, such as collective expulsions, the denial of the right to refuge, and violation of the right of non-refoulment.

In the second set, we found the (in)mobility processes related to the border closure. Border closure policies linked to COVID-19, created in terms of a monocultural rationale, impacted the health of indigenous people and communities. Those policies affected the socio-cultural, economic, and political exchange necessary for social reproduction and protecting life and health. One concealed aspect is the impact these had on the necessary mobility of Andean medical knowledge in terms of elements used and patients and agents of health (such as *Yatiris*, *Qulliris*, and *Usuyiris*). Border closure affected the dynamics of Andean medical knowledge, impacted its cultural integrity, and strengthened its criminalization, contrary to the recognition, protection, and development enshrined by international law.

In the intersection of both processes, militarization also impacted the health of immigrants and indigenous communities, reflected in racism and abuses perpetrated by members of the armed forces, not prepared to deal with this complexity, much less in cross-border and intercultural contexts.

These aspects combined allow us to understand why measures that are supposed to protect public health end up producing negative impacts on the health of certain groups and peoples, for they lead us to verify once again that policies have a differential impact on the population, according to the structural vulnerability involved. Furthermore, they also invite us to think about who pays the price for the defense of public health when health-related arguments give space and legitimacy to the securitizing and racist tendencies still present in the state structure of a country such as Chile.

Our research poses specific challenges: further study of health (im)mobility is needed. Previous work has focused chiefly on health mobility, neglecting the dialectical processes of immobility that the pandemic brought to the forefront. As we do here, those studies may embrace medical pluralism to analyze the effects on health from the point of view of the different medical knowledge present in the territories, surpassing the medical monism that assumes biomedicine is the only worthy knowledge. Finally, more research is needed to show the gap between what the legislation establishes and what the States do. This research will help us shed more light on the permanent contradiction of States that consider themselves democratic, but based on their neoliberal matrix, permanently and selectively reproduce structural violence against populations such as indigenous and immigrants.

## Figures and Tables

**Table 1:** List of immigrants interviewed.

Actor	Gender	Nationality	Age	Residence	Date	Interview Modality
Immigrant	Female	Bolivian	Adult	Iquique (Chile)	apr-21	Virtual
Immigrant	Male	Bolivian	Adult	Iquique (Chile)	apr-21	In person
Immigrant	Female	Bolivian	Adult	Iquique (Chile)	may-21	In person
Immigrant	Female	Bolivian	Adult	Iquique (Chile)	apr-21	In person
Immigrant	Female	Bolivian	Adult	Iquique (Chile)	mar-21	In person
Immigrant	Female	Bolivian	Adult	Iquique (Chile)	apr-21	In person
Immigrant	Male	Bolivian	Adult	Iquique (Chile)	may-21	Virtual
Immigrant	Female	Bolivian	Adult	Iquique (Chile)	may-21	In person
Immigrant	Male	Bolivian	Adult	Iquique (Chile)	may-21	In person
Immigrant	Female	Colombian	Adult	In transit	nov-21	In person
Immigrant	Male	Colombian	Elderly	In transit	nov-21	In person
Immigrant	Male	Colombian	Elderly	In transit	nov-21	In person
Immigrant	Male	Colombian	Young	In transit	nov-21	In person
Immigrant	Female	Colombian	Elderly	In transit	nov-21	In person
Immigrant	LGBTQ+	Cuban	Adult	In transit	nov-21	In person
Immigrant	Female	Ecuadorian	Adult	In transit	nov-21	In person
Immigrant	Female	Venezuelan	Young	In transit	nov-21	In person
Immigrant	Female	Venezuelan	Young	In transit	nov-21	In person
Immigrant	Male	Venezuelan	Young	In transit	nov-21	In person
Immigrant	Female	Venezuelan	Young	In transit	nov-21	In person
Immigrant	Female	Venezuelan	Young	In transit	nov-21	In person
Immigrant	Male	Venezuelan	Adult	In transit	nov-21	In person
Immigrant	Male	Venezuelan	Young	In transit	nov-21	In person
Immigrant	Female	Venezuelan	Young	In transit	nov-21	In person
Immigrant	Male	Venezuelan	Young	In transit	nov-21	In person
Immigrant	Male	Venezuelan	Adult	In transit	nov-21	In person
Immigrant	Female	Venezuelan	Adult	In transit	nov-21	In person
Immigrant	Female	Venezuelan	Adult	In transit	nov-21	In person
Immigrant	Female	Venezuelan	Adult	In transit	nov-21	In person
Immigrant	Female	Venezuelan	Adult	In transit	nov-21	In person
Immigrant	Female	Venezuelan	Young	In transit	nov-21	In person
Immigrant	Female	Venezuelan	Young	In transit	nov-21	In person
Immigrant	Male	Venezuelan	Adult	In transit	nov-21	In person
Immigrant	Male	Venezuelan	Adult	In transit	nov-21	In person
Immigrant	Female	Venezuelan	Adult	In transit	nov-21	In person
Immigrant	Male	Venezuelan	Adult	In transit	nov-21	In person
Immigrant	Female	Venezuelan	Young	In transit	nov-21	In person
Immigrant	Male	Venezuelan	Adult	In transit	nov-21	In person
Immigrant	Male	Venezuelan	Young	In transit	nov-21	In person
Immigrant	LGBTQ+	Venezuelan	Young	In transit	nov-21	In person
Immigrant	Female	Venezuelan	Young	In transit	nov-21	In person
Immigrant	Female	Venezuelan	Young	In transit	nov-21	In person

**Table 2:** Key informants interviewed.

Actor	Gender	Nationality	Age	Residence	Date	Interview Modality
Yatiri	Male	Peruvian	Elderly	Tacna (Perú)	feb-21	In person
Yatiri	Male	Peruvian	Elderly	Tacna (Perú)	feb-21	In person
Yatiri	Male	Bolivian	Elderly	Alto Hospicio (Chile)	feb-21	In person
Yatiri	Male	Bolivian	Elderly	Oruro (Bolivia)	feb-21	In person
Human Rights Lawyer	Male	Chilean	Adult	Iquique (Chile)	dic-21	In person
Church Volunteer	Male	Chilean	Adult	Iquique (Chile)	mar-22	In person
Immigrant leader	Female	Bolivian	Adult	Iquique (Chile)	apr-21	In person
Psychologist	Female	Chilean	Adult	Iquique (Chile)	feb-22	In person
Lawyer NGO	Female	Chilean	Adult	Iquique (Chile)	may-22	In person
